# Identification and Isolation of an Intermediate Metabolite with Dual Antioxidant and Anti-Proliferative Activity Present in the Fungus *Antrodia cinnamomea* Cultured on an Alternative Medium with *Cinnamomum kanehirai* Leaf Extract

**DOI:** 10.3390/plants10040737

**Published:** 2021-04-09

**Authors:** Wen-Wen Zeng, Tsan-Chi Chen, Cheng-Huan Liu, Sheng-Yang Wang, Jei-Fu Shaw, Yu-Ting Chen

**Affiliations:** 1 Institute of Genomics and Bioinformatics, National Chung Hsing University, Taichung 40227, Taiwan; wenwen1116@gmail.com (W.-W.Z.); pudding1714@hotmail.com (C.-H.L.); 2 Department of Ophthalmology, Far Eastern Memorial Hospital, Banqiao District, New Taipei City 22060, Taiwan; 960887272@mail.femh.org.tw; 3 Department of Food Science and Biotechnology, National Chung Hsing University, Taichung 40227, Taiwan; 4 Department of Forestry, National Chung Hsing University, Taichung 40227, Taiwan; taiwanfir@dragon.nchu.edu.tw; 5 Department of Biological Science & Technology, I-Shou University, Kaohsiung 84001, Taiwan; shawjf@isu.edu.tw; 6 Ph.D. Program in Medical Biotechnology, National Chung Hsing University, Taichung 40227, Taiwan

**Keywords:** *Antrodia cinnamomea*, 2,4-dimethoxy-6-methylbenzene-1,3-diol, antioxidant, anti-proliferative, *Cinnamomum kanehirai*

## Abstract

The fungus *Antrodia cinnamomea* has been used as a folk medicine for various diseases, especially cancer. When *A. cinnamomea* is cultured on the original host, an endangered woody plant *Cinnamomum kanehirai* Hayata, the fungus produces more active ingredients, but its growth is slow. Here, *C. kanehirai* leaf ethanol extract (KLEE) was used as a substitute for *C. kanehirai* wood to culture *A. cinnamomea* on solid medium to shorten the culture period and produce active metabolites en masse. The antioxidant activities of methanol extracts from *A. cinnamomea* cultured on KLEE (MEAC-KLEE) were evaluated by 2,2-diphenyl-1-picrylhydrazyl (DPPH) radical-scavenging effect, reducing power, and ferrous ion-chelating effect, and the effective concentration (EC_50_) values were 0.27, 0.74, and 0.37 mg mL^−1^, respectively. MEAC-KLEE exhibited specific anti-proliferative activity against a non-small-cell lung cancer cell line (A549) by Annexin V assay. A secondary metabolite (2,4-dimethoxy-6-methylbenzene-1,3-diol, DMMB) present in the extract (MEAC-KLEE) was purified by high-performance liquid chromatography (HPLC) and identified by nuclear magnetic resonance (NMR) spectra. DMMB exhibited moderate antioxidant activity against DPPH radicals and reducing power, with EC_50_ values of 12.97 and 25.59 μg mL^−1^, respectively, and also induced apoptosis in A549 cells. Our results provide valuable insight into the development of DMMB for nutraceutical biotechnology.

## 1. Introduction

*Antrodia cinnamomea*, known as AC fungus, and in Taiwanese “*Niu-chang-chih*”, is a saprophytic fungus in the Polyporaceae family. Recently, *A. cinnamomea*, especially its fruit body, has been used as a folk medicine for the treatment of various diseases, including liver diseases, abdominal pain, and food intoxication [1,2]. Wild *A. cinnamomea* grows slowly, and its fruit body is only found on the trunk of the aromatic camphor tree *Cinnamomum kanehirai* Hayata, which is endangered and native to Taiwan [3]. Because of its rareness and difficult cultivation, *A. cinnamomea* is expensive and scarce, analogous to the “ruby in Taiwanese forests” [4]. 

Extracts of *A. cinnamomea* have exhibited anti-cancer activity against hepatoma, leukemia, oral cancer, bladder cancer, non-small-cell lung cancer, and colon cancer [5,6,7,8,9,10]. Many ingredients isolated from *A. cinnamomea*, such as polysaccharides, triterpenoids, phenyl compounds, lactones, steroids, adenosine, and ergosterol, have various pharmacological functions, including antioxidant, anti-inflammation, immunity-increasing, and anti-hepatitis functions [2,9,10]. The antioxidant activities of the methanol extracts (MEs) from *A. camphorata* could be attributable to phenolic compounds [11]. For example, 5-methyl-benzo [1,3]-dioxole-4,7-diol, a phenolic compound, purified from *A. cinnamomea* culture filtrate, exhibits antioxidant activity against 2,2-diphenyl-1-picrylhydrazyl (DPPH), a free radical [12]. In addition, 4,7-dimethoxy-5-methyl-l,3-benzodioxole, isolated from the fruiting body of *A. cinnamomea*, can inhibit the proliferation of colon adenocarcinoma cell lines [13]. 

Production of active compounds by *A. cinnamomea* is greater when it is cultured on the original host (i.e., *C. kanehirai*) than when cultured on other cultural woods (media) [14]. However, the time required for traditional culture is long, and the precious and endangered *C. kanehirai* is scarce. Increasing the demand for *C. kanehirai* would result in ecological damage. To avoid this increased demand for *C. kanehirai* wood and to shorten the culture period, we examined whether *C. kanehirai* leaf extract could replace *C. kanehirai* wood for culturing *A. cinnamomea*. In this study, we determined the effects on phenolic compounds production by *A. cinnamomea* cultured with different *C. kanehirai* Hayata leaf extracts, including water extract (JTA-KLWE), ethanol extract (JTA-KLEE), and flora water (JTA-KFW). To date, studies on extracts of *A. cinnamomea* have focused on either its anti-proliferative or antioxidant activities, we examined both. The antioxidant and anti-proliferative activities of MEs from mycelia, inoculated medium (in-medium) of *A. cinnamomea* were determined. Furthermore, the main phenolic compound that contributed to the major antioxidant and anti-proliferative capacity in the MEs from *A. cinnamomea* was isolated and analyzed.

## 2. Results

### 2.1. Comparisons of Mycelial Growth and Phenolic Compound Content of MEs from A. cinnamomea in Various Media

Because wild *A. cinnamomea* is found only on the trunk of the aromatic tree *Cinnamomum kanehirai* Hayata, this tree may provide essential elements for *A. cinnamomea* growth. To decrease ecological damage and to shorten the culture period, we attempted to use *C. kanehirai* leaf water extract (KLWE), *C. kanehirai* leaf ethanol extract (KLEE), *C. kanehirai* flora water (KFW) and other chemicals, such as squalene, the precursor of triterpenoids [15], and chitosan, which can be used to increase the triterpenoid content [16], as substitutes for *C. kanehirai* wood to culture *A. cinnamomea* on solid medium, and their effects on the growth and bioactivity of *A. cinnamomea* mycelia were also investigated.

**Figure 1:**
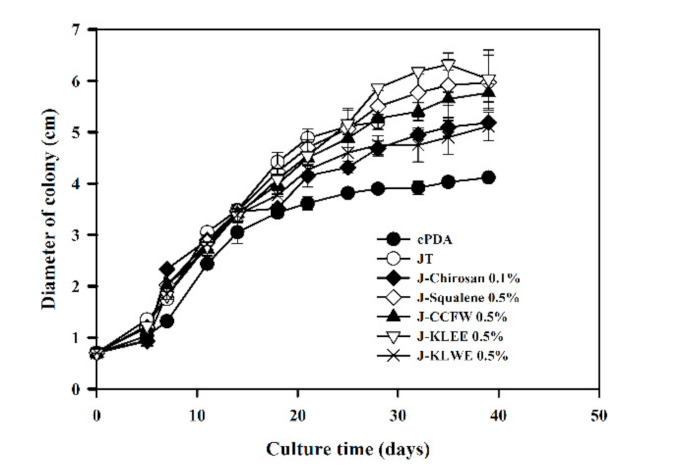
Growth curves for *A. camphorata* cultured on various media. Each value is expressed as the mean ± SD (*n* = 3).

**Figure A1:**
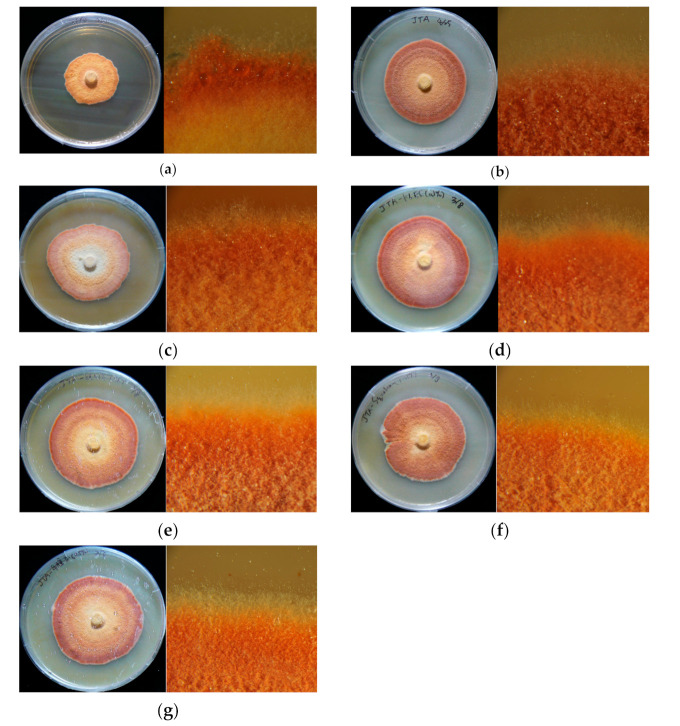
Morphology of colonies of *A. camphobrata* ACT4. *A. camphobrata* ACT4 was cultured on (**a**) cPDA medium for 29 days, (**b**) JTA medium for 29 days, (**c**) JTA-squalene medium for 32 days, (**d**) JTA-KLEE medium for 32 days, (**e**) JKLWE medium for 32 days, (**f**) J KFW medium for 32 days, or (**g**) JTA-chitosan medium for 39 days. The morphology of colonies was showed on the left and the view under a stereomicroscope (20×) was showed on the right.

After culturing for 29 days, the growth of mycelia began to slow down (Figure 1), and the color of the mycelia changed from red to orange (Figure A1). The mycelia cultured in all Job’s tears agar (JTA) media (i.e., JTA-squalene, JTA-chitosan, JTA-KLEE, JTA-KLWE, JTA-KFW, and JTA-chitosan) exhibited a similar color-change phenomenon after 32 days. Therefore, the mycelia were harvested at day 32. The mycelia grew best on JTA-KLEE (6.18 ± 0.06 cm) (Figure 1). This finding indicates that the additional ethanol extract of the *C. kanehirai* leaf promoted the growth of mycelia of *A. cinnamomea*.

The rapid microplate Folin–Ciocalteu assay was employed to determine the total phenolic contents of methanol extracts (MEs) from *A. cinnamomea*. As shown in Table 1, the extract from the *A. cinnamomea* in-medium contained more phenolic compounds (approximately 110–165 gallic acid equivalents (GAE) mg g^−1^) than the extract from *A. cinnamomea* mycelia (49–61 GAE mg g^−1^). In other words, *A. cinnamomea* produced more phenolic compounds and secreted them into the medium. The addition of KLEE, KLWE, and KLWE effectively increased the production of phenolic compounds in mycelia and in-medium. The production of phenolic compounds in KLEE increased by approximately 20% in both the mycelia and the secretion.

### 2.2. Antioxidant Activity of MEs from A. cinnamomea

**Figure 2:**
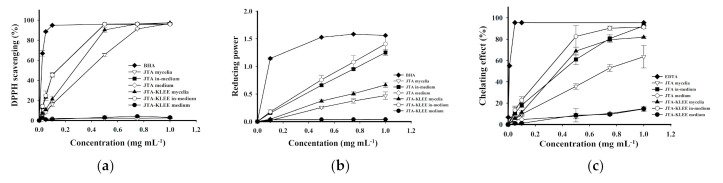
Antioxidant activity assays of MEs of *A. cinnamomea* mycelia, inoculated medium (in-medium), and fresh medium. (**a**) DPPH free radical-scavenging effects. (**b**) Reducing power of ferrous ions. (**c**) Ferrous ion-chelating effects. The MEs of *A. cinnamomea* mycelia from cultured on JTA. Each value is expressed as the mean ± SD (*n* = 3).

Shu and Lung have reported that the total phenolic compounds were primarily responsible for the antioxidant activity of *A. cinnamomea* [11]. The results in Table 1 demonstrate that *A. cinnamomea* cultured in JTA-KLEE produced the highest level of phenolic compounds. We thus investigated the influence of addition of KLEE to the medium of *A. cinnamomea* mycelia on the antioxidant activity. To identify the materials present the antioxidant effects are the extracts of the fungus or those from the ethanol extracts of the leaf, we analyzed the MEs of mycelia, in-medium, or fresh medium to represent the bioactive compound produced by *A. cinnamomea*, secreted from *A. cinnamomea*, or from the leaf. The 2,2-diphenyl-1-picrylhydrazyl (DPPH)-scavenging ability increased as the concentration of the extract increased from 0 to 1 mg mL^−1^ and reached maxima at 0.5, 0.5, 1.0, and 0.75 mg mL^−1^ of JTA in-medium, JTA-KLEE in-medium, JTA mycelia, and JTA-KLEE mycelia, respectively (Figure 2). At 0.5 mg mL^−1^, the scavenging effects were 66%, 90%, 96%, and 96% for JTA mycelia, JTA-KLEE mycelia, JTA in-medium and JTA-KLEE in-medium, respectively. The high scavenging effect of both in-media were similar to that of BHA (96%) on the scavenging of DPPH radicals. JTA in-medium and JTA-KLEE in-medium exhibited higher DPPH-scavenging activities (EC_50_ = 0.14 and 0.14 mg mL^−1^, respectively) than *A. cinnamomea* mycelia cultured on JTA and JTA-KLEE medium (EC_50_ = 0.37 and 0.27 mg mL^−1^, respectively) (Table 2). Our findings indicate that some metabolites with DPPH-scavenging effects produced by *A. cinnamomea* mycelia can be significantly increased by *C. kanehirai* leaf ethanol extract.

The reducing power of the ME was also assayed. The results in Figure 2b, indicated that both JTA in-medium and JTA-KLEE in-medium exhibited significantly higher reduction capacities than JTA mycelia and JTA-KLEE mycelia. At a concentration of 0.75 mg mL^−1^, the reducing powers of the ME were 0.95 from the JTA in-medium and 1.08 from the JTA-KLEE in-medium, whereas the reducing powers were 0.37 and 0.51 from JTA mycelia and JTA-KLEE mycelia, respectively. The effective concentration for 50% reduction capacity was calculated, and the EC_50_ values of the mycelia were 1.05 and 0.74 mg mL^−1^ on JTA and JTA-KLEE, respectively (Table 2). 

The ferrous ion-chelating effects of the MEs from all of the *A. cinnamomea* mycelia and in-media are shown in Figure 2c. The chelating effects were increased as the concentration increased from 0.025 to 1.0 mg mL^−1^. For ME at a concentration of 0.5 mg mL^−1^, the chelating abilities from the JTA mycelia, JTA-KLEE mycelia, JTA in-medium and JTA-KLEE in-medium were 36%, 69%, 61% and 83%, respectively. As calculated, the EC_50_ values for ferrous ion chelating of MEs from JTA mycelia, JTA-KLEE mycelia, JTA in-medium and JTA-KLEE in-medium were 0.71, 0.37, 0.4, and 0.29 mg mL^−1^, respectively.

### 2.3. Cellular Effects of MEAC-KLEE on Cancer Cells

**Figure 3:**
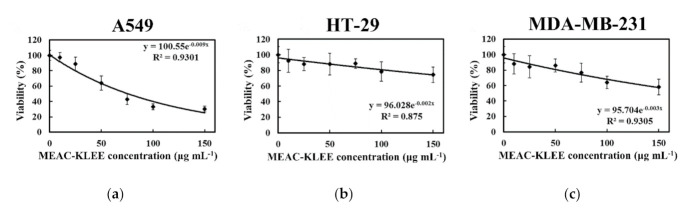
Effects of MEAC-KLEE on cancer cells. After treated with MEAC-KLEE, the viabilities of (**a**) A549, (**b**) HT-29, and (**c**) MDA-MB-231 cells were analyzed according to the formazan-based cell proliferation reagent WST-1 assay. Each value is expressed as the mean ± SD (*n* = 3).

To explore the effect of MEAC-KLEE on cancer cells, A549 (human non-small-cell lung cancer) cells, HT-29 (human colon cancer) cells, and MDA-MB-231 (breast cancer) cells were incubated with MEAC-KLEE, respectively. The WST-1 assay indicated that treatment with MEAC-KLEE (150 μg mL^−1^) inhibited the viability of A549, HT-29, and MDA-MB-231 cells by 70%, 26%, and 42%, respectively (Figure 3). MEAC-KLEE (150 μg mL^−1^) significantly reduced the A549 cell viability, with an IC_50_ value of 77.6 μg mL^−1^ (Figure 3a). However, MEAC-KLEE exhibited lower cytotoxicity against HT-29 and MDA-MB-231 (IC_50_ > 150 μg mL^−1^ for both). Thus, the potential anti-proliferative activities MEAC-KLEE’s are more specific to A549 cells than the other two cancer cells.

To determine whether MEAC-KLEE enhanced cell death by inducing cell apoptosis, A549 cells were stained with Annexin V and PI to analyze the apoptotic status (Table 3). Compared with untreated A549 cells, MEAC-KLEE induced significant apoptosis of A549 cells in a concentration-dependent manner (31% apoptotic cells in 200 μg mL^−1^). 

### 2.4. Identification of the Major Compound in MEAC-KLEE

**Figure 4:**
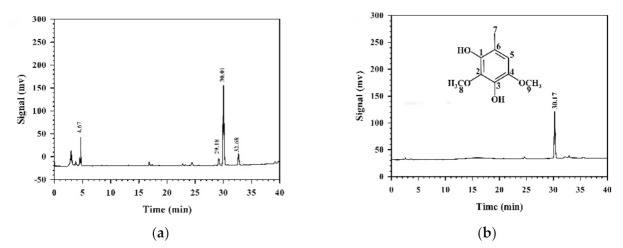
HPLC fingerprints of MEAC-KLEE (**a**) and its purified fraction (**b**) on an SPE C18 column. The chemical structure of the main peak was identified as DMMB (MW 170) based on ^1^H-NMR, ^13^C-NMR, and mass spectra.

To identify the major compound in MEAC-KLEE that contributed to the antioxidant and anti-proliferative activities, the high-performance liquid chromatography (HPLC) fingerprint was analyzed. The major compound in MEAC-KLEE appeared on HPLC at a retention time of 30 min (Figure 4a). This compound was purified using an SPE C18 column, and its chemical structure was identified as 2,4-dimethoxy-6-methylbenzene-1,3-diol (DMMB) based on nuclear magnetic resonance (NMR) and mass spectra (Figure 4b). DMMB: ^1^H-NMR (400 MHz, CDCl_3_): 6.38 (1H, s, H-5), 3.94 (3H, s, OCH_3_-2), 3.80 (3H, s, OCH_3_-4), 2.17 (3H, s, CH_3_-6); ^13^C-NMR (100 MHz, CDCl_3_): 141.3 (C-1), 140.3 (C-4), 136.2 (C-3), 134.1 (C-2), 113.4 (C-6), 108.1 (C-5), 60.7 (OCH_3_-2), 56.8 (OCH_3_-4), 15.2 (CH_3_-6); ESIMS: *m*/*z* = 183 [M-H]^−^. HRESIMS: *m*/*z* = 183.0652 [M-H]^−^ (calculated for C_9_H_11_O_4_, 183.0657). These characteristics are similar to reported values [17].

### 2.5. Antioxidant Activity Assay of DMMB

**Figure 5:**
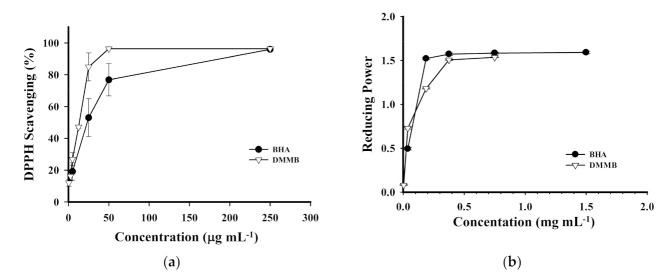
Antioxidant activity assays of DMMB. (**a**) DPPH free radical-scavenging effects. (**b**) Reducing power. Each value is expressed as the mean ± SD (*n* = 3).

DMMB may be a powerful antioxidant. Its structure contains two hydroxyl groups on the benzene ring, which can be used to bind radicals along with the benzene ring. The resonance structure can stabilize the free electrons. To determine whether the antioxidant activity of MEAC-KLEE was attributable to DMMB, its DPPH-scavenging capacity, reducing power, and ferrous ion-chelating ability were examined. DMMB exhibited a higher DPPH-scavenging ability (EC_50_ = 12.97 μg mL^−1^) than BHA (EC_50_ = 23.22 μg mL^−1^) (Figure 5a). In addition, its reducing power was better than that of BHA at a low concentration (Figure 5b). The effective concentrations for 50% reduction (EC_50_) of DMMB and BHA were 25.59 and 38.42 μg mL^−1^, respectively. However, the ferrous ion-chelating ability was not detected using DMMB, even at a concentration of 1 mg mL^−1^. Our results suggest that DMMB is the primary compound responsible for the DPPH-scavenging ability and reducing power, but other compounds must be responsible for the chelating ability of MEAC-KLEE that is worthy of further study.

### 2.6. Cellular Effects of DMMB on Cancer Cells

**Figure 6:**
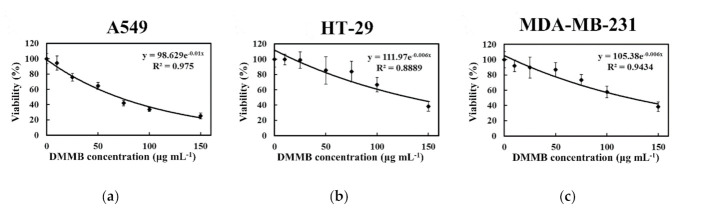
Effects of DMMB on cancer cells. After treated with DMMB, the viabilities of (**a**) A549, (**b**) HT-29, and (**c**) MDA-MB-231 cells were analyzed according to the formazan-based cell proliferation reagent WST-1 assay. Each value is expressed as the mean ± SD (*n* = 3).

The cytotoxicity of DMMB on cancer cells was also determined (Figure 6). The WST-1 assay revealed that DMMB reduced the tumor cell number in a concentration-dependent manner. DMMB significantly reduced the viability of A549, HT-29, and MDA-MB-231 cells, with IC_50_ values of 67.9, 134.4, and 124.3 μg mL^−1^, respectively. 

Compared with untreated A549 cells (9% apoptosis), DMMB significantly induced apoptosis of the A549 cells in a concentration-dependent manner (82% apoptotic cells with 200 μg mL^−1^) (Table 4). DMMB treatment was more effective than MEAC-KLEE (31% apoptosis). The IC_50_ values for pure DMMB and MEAC-KLEE were 67.9 μg mL^−1^ (Figure 6a) and 77.6 μg mL^−1^ (Figure 3a), respectively. This finding suggests that DMMB may be the primary compound in MEAC-KLEE that contributes to cell toxicity by inducing apoptosis. 

## 3. Discussion

*C. kanehirae* Hayata is a broadleaf camphor tree and the native host of medical fungus *A. cinnamomea.* Camphor oil was traditionally used as a fumigant during the era of the Black Death [18], and more recently, *C. kanekirae* twig essential oils have a specific antibacterial efficacy against *V. alginolyticus* from farmed aquatic white shrimp [19]. The ethanol extracts of *C. kanehirai* leaves exhibit anti-cancer activity by inducing apoptosis in human hepatoma HA22T/VGH and HepG2 cells through caspase-3 cascade [20], however, their IC_50_ were 0.25 and 0.76 mg mL^−1^, respectively. It’s a high IC_50_ value for anti-cancer drug development. 

*A. cinnamomea*, an infected fungus of *C. kanekirae*, has been used as a folk medicine for the treatment of various diseases, including cancers. Based on the evaluation of the ergostane-type triterpenoids and polyacetylenes, which possess good anti-inflammation activity, the production of active compounds by *A. cinnamomea* is greatest when it is cultured on *C. kanehirai* [14]. Thus people hew this tree to harvest or cultivate this medical fungus, leading to the endangerment of *C. kanehirai* and ecological damage. The trees not only produce the active compound but also provide the substrates to affect the metabolites synthesis of parasites. We used *C. kanehirai* leaf ethanol extract as an ingredient in the *A. cinnamomea* cultured medium, which could promote growth (Figure 1), phenolic compounds production (Table 1), and antioxidant activity of mycelia (Figure 2). 

The total phenolic contents of the MEs of *A. cinnamomea* mycelia (49–61 mg g^−1^) found in this study were higher than those in the methanol, ethyl acetate, and hexane extracts (38, 16, and 12 mg g^−1^, respectively) of *A. cinnamomea* mycelia as reported by Song and Yen [21]. Similarly, the yield of total phenolic compounds (110–165 mg g^−1^) from the MEs of the in-medium in this study was 1.6- to 2.2-fold of that from the dry matter of the filtrate (DMF) of the mycelial extracellular medium (67 mg g^−1^) as reported by Song and Yen [21]. Shu and Lung have also reported that the scavenging effects of DPPH radicals by the MEs (0.6 mg mL^−1^) of *A. cinnamomea* filtrates (75%) were higher than that of the mycelia (65%) [11]. The EC_50_ values for reducing the power of the mycelia were 1.05 and 0.74 mg mL^−1^ on JTA and JTA-KLEE, respectively (Table 2). These values are significantly lower than those reported by Mau et al. [22], whose EC_50_ values for the reducing power of red and white mycelia were 2.00 and 1.56 mg mL^−1^, respectively. It has been reported that the EC_50_ values for the chelating effect of red and white mycelia were 1.21 and 1.78 mg mL^−1^, respectively [22], and those of white mycelia and fruit bodies were 2.19 and 1.77–3.54 mg mL^−1^, respectively [23]. MEs from *A. cinnamomea* mycelia and in-medium in the present study exhibited higher ferrous ion-chelating effects (with EC_50_ values of 0.37 and 0.29 mg mL^−1^, respectively) (Table 2) than were observed in these previous studies, indicating that the *C. kanehirai* leaf ethanol extract significantly increased the chelating ability.

In general, culturing *A. cinnamomea* on JTA solid culture medium with additives increased the production of bioactive antioxidants, as shown by increased DPPH-scavenging effects, reducing power, and ferrous ion-chelating abilities. These findings (Table 2) clearly show that the addition of the *C. kanehirai* leaf ethanol extract increased the antioxidant activity, and they suggest that the *C. kanehirai* leaves can be used to replace wood for culturing *A. camphorata*, thereby reducing costs and saving the endangered *C. kanehirai.* In addition, the effective antioxidant concentration (EC_50_) of the *A. camphorata* in-medium was significantly lower than that of the mycelia. Given the absent DPPH scavenging, reducing power, and ferrous ion-chelating effects of the fresh media, the compounds with antioxidant activity of the in-medium was not derived from the culture medium but secreted from *A. cinnamomea* mycelia [11,21]. Our finding indicated that the in-medium also has the potential for the production of antioxidation products as dietary supplements.

The DMMB was the major compound isolated from MEAC-KLEE (Figure 4a), which had been reported to alleviate atopic dermatitis and inhibit the growth of methicillin-resistant *Staphylococcus aureus* (MRSA) by playing a role as a DNA polymerase inhibitor [24]. The in vitro cytotoxicity against HaCaT study showed no cytotoxicity of applied DMMB at the concentrations of 10 and 50 μg mL^−1^ and over 90% viability of the does up to 100 μg mL^−1^, and the in vivo tolerance study demonstrated no irritation of topically applied DMMB at 1 mg mL^−1^ [24]. Thus topical DMMB application manifested a minimal adverse event on normal skin [24]. Considering its in vivo safety and function on antioxidant and inhibition of DNA replication, the effects of DMMB on cancer cells were investigated in this study. Compared with the IC_50_ values for pure DMMB (Figure 6a; 67.9 μg mL^−1^) and MEAC-KLEE (Figure 3a, 77.6 μg mL^−1^), DMMB was suggested as the primary compound in MEAC-KLEE that contributes to cell toxicity by inducing apoptosis in the A549 cell line. For the high IC_50_ value of DMMB, the structure modification is necessary for anti-cancer drug development. However, based on its moderate antioxidant activity against DPPH radicals and reducing power, DMMB has the potential to develop as a nutraceutical for scavenging ROS to prevent activation of pro-tumorigenic signaling pathways [25].

## 4. Materials and Methods

### 4.1. Plant Material and Preparation of C. kanehirai Leaf Extract

The leaves of *C. kanehirae* were collected in May 2015 from Nantou County, Taiwan, and were identified by Professor Sheng-Yang Wang (NCHU). The voucher specimen was deposited in the herbarium of the same university. The *C. kanehirai* leaf water extract (KLWE) or ethanol extract (KLEE) was prepared by blending 5 g of *C. kanehirai* leaves with 100 mL of deionized water or ethanol (Merck, Darmstadt, Germany), respectively. After sonication at a frequency of 40 kHz for 1 h, the mixture was centrifuged at 1000 rpm for 10 min and filtered through gauze. The KLWE was sterilized at 121 °C for 20 min. The KLEE was concentrated using a rotary evaporator at 40 °C to a tenth of the original volume and then filtered (0.2 µm). Both extracts were stored at room temperature.

### 4.2. Fungal Strain and Medium Preparation

*A. camphorata* ACT4, obtained from Professor Sheng-Yang Wang (NCHU) was cultured on potato dextran agar (PDA, BD, Spark, MD, USA) or Job’s tears agar (JTA) medium at 28 °C. The JTA medium was prepared by mixing 4 g of brown Job’s tears powder, 4 g of glucose (Sigma-Aldrich, St. Louis, MO, USA), 0.2 g of peptone (USB Corp., Cleveland, OH, USA) and 3 g of agar in 200 mL of deionized water. To 200 mL of the sterilized JTA medium, 1 mL of the *C. kanehirai* leaf water extract (JTA-KLWE), ethanol extract (JTA-KLEE), *C. kanehirai* flora water (JTA-KFW) (Marchese Bio-tech, Nantou, Taiwan), or squalene (JTA-Squalene) (Sigma-Aldrich) was added. In addition, 0.2 g of chitosan (JTA-Chitosan) (Sigma-Aldrich) was added to 200 mL of the JTA medium prior to sterilization.

### 4.3. Preparation of the Methanol Extracts (MEs) of A. camphorata ACT4

After culture for 29 days, the mycelia and medium were separated by scraping. Ten milliliters of methanol (Merck) was mixed with 0.9 g *A. camphorata* ACT4 mycelia, *A. camphorata* ACT4 in-medium, or fresh culture medium (control) and then sonicated for 1 h. After filtration through Whatman No. 4 filter paper, the extracts were dried at 40 °C with nitrogen. The dried MEs were dissolved in 80% (*v*/*v*) methanol in 25 mg mL^−1^ for further experiments.

### 4.4. Determination of the Total Phenolic Compound Content

The rapid microplate Folin-Ciocalteu assay for the phenolic compounds was performed using an Infinite F200 plate reader (Tecan Trading AG, Zürich, Switzerland) [26]. Fifty microliters of the sample was mixed with 50 µL of the Folin-Ciocalteu reagent (diluted 1:5, *v*/*v*) (Sigma-Aldrich) in each well, followed by the addition of 100 µL of 0.35 M sodium hydroxide solution. The formed blue complex was measured at 750 nm. Gallic acid (Sigma-Aldrich) was used as a standard. The total phenolic content was expressed as milligrams of gallic acid equivalents (GAE) per gram (dw) of ME.

### 4.5. Purification of the Phenyl Compound

The separation of phenolic acids of the ME was performed with an HPLC (HITACHI L-6200, Hitachi High Technologies Com., Tokyo, Japan) system with a Syncronis C18 column (5 μm, 4.6 mm × 250 mm; Thermo Fisher Scientific Inc., Waltham, MA, USA). The mobile phase (1.0 mL min^−1^) consisted of A: 0.1% formic acid (FA; Thermo Fisher Scientific Inc., Rockford, IL, USA); and B: 100% methanol. The gradient conditions (A:B) were programmed as 0–10 min, 5–25% A and 95–75% B; 10–20 min, 25–30% A and 75–70% B; 20–40 min, 30–60% A and 70–40% B. The column was maintained at 25 °C, and the eluents were detected by UV280. Instrument control and data analysis were conducted using ECL2000 software (Analab Corp., Taipei, Taiwan).

The functional phenyl compound 2,4-dimethoxy-6-methylbenzene-1,3-diol (DMMB) was purified by preparative HPLC (Primeline^TM^ Gradient pump 500G; Analytical Scientific Ins., El Sobrante, CA, USA). The DMMB was separated on a preparative Inspire^TM^ C18 column (21.2 mm × 250 mm; Dikma Technologies Inc., Lake Forest, CA, USA) with 40% methanol (10.4 mL min^−1^). The fraction containing DMMB was detected by UV280 and collected using an Advantec SF2120 fraction collector (Advantec Toyo Kaisha, Ltd., Tokyo, Japan) simultaneously. The collected fractions were concentrated using a rotary evaporator and a pressured nitrogen gas-blowing concentrator.

### 4.6. Structural Analysis of DMMB

The ^1^H and ^13^C nuclear magnetic resonance (NMR) spectra were recorded on a Bruker AVANCEIII 400 (Ettlingen, Germany). The low- and high-resolution mass spectra were obtained using a Finnigan LCQ ion-trap mass spectrometer (Finnigan Corporation, San Jose, CA, USA) and LTQ Orbitrap XL (Thermo Fisher Scientific Inc., Rockford, IL, USA), respectively.

### 4.7. Determination of DPPH-Scavenging Activity

The scavenging ability of MEs on DPPH (Sigma-Aldrich) was analyzed using a modification of the method described by Shimada et al. [27]. One hundred microliters of the ME sample in methanol was mixed with 300 µL of 0.1 mM DPPH, and they were shaken in the dark for 30 min. The degree of discoloration was measured at 517 nm against a blank. Butylated hydroxyanisole (BHA) (Sigma-Aldrich) was used as the standard. The percentage of DPPH scavenged (DPPHsc) was calculated as follows:

(1) $${\mathrm{DPPH}}_{\mathrm{SC}}\;(\%)\;=\;\frac{\left[A_{c}-\left(A-A_{b}\right)\right]}{A_{c}}\times 100,$$

where A_c_ is the absorbance of the control, A is the absorbance of the sample or standard, and A_b_ is the original color of the sample. EC_50_ (mg mL^−1^), defined as the effective concentration when 50% of the DPPH free radicals were scavenged, was determined by interpolation. 

### 4.8. Ferric-Reducing Antioxidant Power Assay

The ferric-reducing power was determined using a method reported by Oyaizu [28] with modification. One hundred microliters of the ME in 80% methanol was mixed with 100 µL of 0.2 M sodium phosphate buffer (pH 6.6) and 100 µL of 1% (*w*/*v*) potassium ferricyanide (Sigma-Aldrich), and then incubated at 50 °C for 20 min. After cooling, 100 µL of 10% (*w*/*v*) trichloroacetic acid (Sigma-Aldrich) was added. The mixture was centrifuged at 200 rpm for 10 min. Then, 250 µL of the upper layer was mixed with 250 µL of deionized water and 50 µL of 0.1% (*w*/*v*) ferric chloride. After the reaction for 10 min, the OD700 value was measured to indicate reducing power. EC_50_ (mg mL^−1^) was defined as the effective concentration when the absorbance was 0.5 and was estimated via interpolation. BHA was used as the control.

### 4.9. Ferrous Ion-Chelating Assay

The ferrous ion-chelating ability was determined using the method described by Decker and Welch [29] with modification. Two hundred microliters of the ME was mixed with 30 µL of 0.35 mM ferrous chloride (Fluka Chemie AG, Buchs, Switzerland) and then 30 µL of 1.7 mM FerroZine^TM^ (Sigma-Aldrich), and the mixture was allowed to react for 10 min. The absorbance at 562 nm (A) was measured against a blank (A_B_), and the ferrous ion--chelating activity of the extracts was calculated as follows:

(2) $$\mathrm{ferrous}\;\mathrm{ion}-\mathrm{chelating}\;\mathrm{activity}\;(\%)\;=\;\frac{A_{B}-A}{A_{B}}\times 100,$$

A lower absorbance indicates a higher ferrous ion-chelating effect. EC_50_ (mg mL^−1^), defined as the effective concentration when 50% of the ferrous ions were chelated, was obtained via interpolation. 

### 4.10. Cell Culture

Three cell lines, A549 (CCL-185; ATCC, Manassas, VA, USA), HT-29 (HTB-38; ATCC), and MDA-MB-231 (CRM-HTB-26; ATCC), were cultivated in Dulbecco’s modified Eagle’s medium (Thermo Fisher Scientific Inc., Rockford, IL, USA) containing 10% fetal bovine serum (Biological Industries, Kibbutz Beit Haemek, Israel). Cells (1 × 10^6^) were seeded in 6-cm cell culture dishes and cultured in a humidified incubator at 37 °C under 5% CO_2_. 

### 4.11. Cytotoxicity Assay

The cell viabilities were evaluated using a formazan-based assay involving the cell proliferation reagent WST-1 (Roche Applied Science, Mannheim, Germany) according to the manufacturer’s instructions. Briefly, the cells were adjusted to 5.0 × 10^3^ cells in 100 μL of culture medium and cultured in a 96-well plate until complete adhesion was achieved. After replacement with fresh medium containing MEAC-KLEE or DMMB at various concentrations for 2 days, 10 μL of WST-1 was added to each well and incubated in a 37 °C incubator with 5% CO_2_ for 2 h. The absorbance value at 450 nm was measured against a reference value at 690 nm. The cell viability was calculated as the ratio of the optical density values relative to that of the untreated cells. The IC_50_ values were calculated using Microsoft Excel software.

### 4.12. Apoptosis Assay

For the apoptosis assay, the cells were incubated in a culture medium containing MEAC-KLEE or DMMB for 2 days. The harvested cells were stained using the FITC Annexin V Apoptosis Detection Kit with propidium iodide (PI; BioLegend Inc., San Diego, CA, USA), washed twice with cold phosphate-buffered saline and then incubated in 100 μL of Annexin V Binding Buffer with FITC Annexin V and PI for 15 min in the dark. Finally, 400 μL of Annexin V Binding Buffer was added to each sample and immediately analyzed using a MoFlo^TM^ XDP flow cytometer (Beckman Coulter Inc., Brea, CA, USA) with 488 nm excitation and the appropriate bandpass filter (i.e., 525/20 for FITC and 610/10 for PI).

### 4.13. Statistical Analysis

Data are reported as the means ± standard deviation (SD). The significance of differences between group means was calculated by analysis of variance (ANOVA) using Dunnett’s test.

## 5. Conclusions

In addition to *C. kanehirai* wood, the *C. kanehirai* leaf ethanol extract increased the antioxidant activity and the content of bioactive compounds of *A. cinnamomea*. This alternative medium added with *C. kanehirai* leaf extract for the culture of *A. cinnamomea* fungus can reduce demand for *C. kanehirai* wood with potential environmental impact. The mechanism underlying the induction of the active compounds of *A. cinnamomea* by *C. kanehirai* leaf extract warrants further investigation. The active compounds induced by the *C. kanehirai* leaf ethanol extract may be secreted into the in-medium with a lower antioxidant effective concentration (EC_50_) than the mycelia, suggesting that the in-medium has the potential for use as an antioxidant supplement. Recently, a phenolic compound, 2,3,5-trimethoxy-4-cresol, was isolated from the mycelia of *A. cinnamomea* and found to exhibit anti-metastatic activity [30]. In addition, 5-methyl-benzo [1,3]-dioxole-4,7-diol purified from an *A. cinnamomea* culture filtrate was shown to exhibit strong antioxidant activity against DPPH radicals [12]. In summary, we have demonstrated that leaf of *C. kanehirai* can be used as an alternative and ecologically friendly medium additive to promote *A. cinnamomea* growth and DMMB content in culture. We also determined that DMMB, which is a major phenolic compound in MEAC-KLEE, possesses both antioxidant and promising anti-proliferative activities and induced apoptosis in A549 cells. The results of our study provide useful information for the future development of DMMB as a naturally beneficial product.

## Figures and Tables

**Table 1:** Yield and total phenyl compounds in the MEs from *A. cinnamomea* mycelia, inoculated medium (in-medium), and fresh medium.

	Yield of ME (mg g^−1^) ^1^			TP (GAE mg g^−1^ Extract) ^2^		
	Mycelia	in-Medium ^3^	Medium	Mycelia	in-Medium ^3^	Medium
JTA	15.70 ± 0.22 ^c^	18.76 ± 1.00 ^d^	4.41 ± 0.34 ^b^	50.81 ± 2.50 ^c^	121.70 ± 6.96 ^bc^	2.17 ± 0.19 ^b^
JTA-KLEE (0.5%) ^4^	21.22 ± 0.50 ^b^	25.53 ± 0.71 ^b^	4.31 ± 0.29 ^b^	61.03 ± 2.06 ^a^	141.35 ± 5.03 ^ab^	1.97 ± 0.13 ^c^
JTA-KLWE (5%) ^5^	15.83 ± 0.60 ^c^	18.15 ± 0.83 ^d^	5.42 ± 0.48 ^a^	55.49 ± 2.88 ^b^	159.52 ± 11.38 ^a^	2.51 ± 0.14 ^a^
JTA-KFW (0.5%) ^6^	22.91 ± 0.87 ^a^	19.32 ± 1.01 ^d^	3.63 ± 0.14 ^c^	59.70 ± 2.20 ^a^	164.56 ± 24.60 ^a^	2.39 ± 0.18 ^a^
JTA-Squalene (0.5%)	20.92 ± 0.54 ^b^	36.44 ± 2.43 ^a^	4.82 ± 0.53 ^ab^	50.46 ± 1.35 ^c^	110.12 ± 11.70 ^c^	0.63 ± 0.17 ^d^
JTA-Chitosan (0.1%)	14.60 ± 0.36 ^b^	22.93 ± 0.29 ^c^	4.81 ± 0.05 ^ab^	48.97 ± 0.71 ^c^	129.47 ± 3.22 ^bc^	2.05 ± 0.05 ^bc^

^1^ The yield is expressed as milligrams (dw) of ME per gram of fresh mycelia or medium. Each value is expressed as the mean ± SD (*n* = 3). Means with different letters (a, b, c, d) within the same column differ significantly (*p* < 0.05). ^2^ The total phenyl content is expressed as milligrams of gallic acid equivalents (GAE) per gram (dw) of ME. ^3^ Medium was inoculated with *A. cinnamomea* mycelia. ^4^ KLEE: *C. kanehirai* leaf ethanolic extract. ^5^ KLWE: *C. kanehirai* leaf water extract. ^6^ KFW: *C. kanehirai* floral water.

**Table 2:** EC_50_ values for MEs from *A. cinnamomea* mycelia, inoculated medium (in-medium), and fresh medium.

		EC_50_ (mg mL^−1^) ^1^		
		DPPH sc	Reducing Power	Chelating Effect
Mycelia	JTA	0.37 ± 0.01 ^a^	1.05 ± 0.11 ^a^	0.71 ± 0.04 ^a^
Mycelia	JTA-KLEE	0.27 ± 0.02 ^b^	0.74 ± 0.06 ^b^	0.37 ± 0.02 ^b^
In-medium	JTA	0.14 ± 0.01 ^c^	0.37 ± 0.01 ^c^	0.40 ± 0.04 ^b^
In-medium	JTA-KLEE	0.14 ± 0.02 ^c^	0.32 ± 0.04 ^c^	0.29 ± 0.04 ^c^
Medium	JTA	N^D 2^	N^D^	ND
Medium	JTA-KLEE	N^D^	N^D^	ND
BHA		0.02 ± 0.00 ^d^	0.04 ± 0.00 ^d^	-
EDTA		-	-	0.01 ± 0.00 ^d^

^1^ EC_50_, the effective concentration at which 50% of DPPH radicals were scavenged, the absorbance was 0.5 for the reducing power, and 50% of the ferrous ions were chelated, respectively. Each value is expressed as the mean ± SD (*n* = 3). Means with different letters (a, b, c, d) within a column are significantly different (*p* < 0.05). ^2^ Not detected.

**Table 3:** Cytotoxicity of MEAC-KLEE against A549 cells.

A549 Cells	MEAC-KLEE (μg mL^−1^)				
	0	50	100	150	200
Viable cells	93.27 ± 0.30%	88.79 ± 0.69%	89.22 ± 0.19%	85.93 ± 0.53%	64.96 ± 0.46%
Early apoptosis	0.58 ± 0.04%	2.43 ± 0.19%	1.86 ± 0.15%	1.12 ± 0.06%	2.32 ± 0.19%
Late apoptosis	5.24 ± 0.25%	8.18 ± 0.43%	7.94 ± 0.17%	11.64 ± 0.49%	28.96 ± 0.37%
Necrosis	0.92 ± 0.08%	0.61 ± 0.10%	0.99 ± 0.09%	1.31 ± 0.14%	3.77 ± 0.16%

The gated cell population represents the number of apoptotic A549 cells cultured in the medium containing MEAC-KLEE after 2 days. Each value is expressed as the mean ± SD (*n* = 5).

**Table 4:** Cytotoxicity of DMMB against A549 cells.

A549 Cells	DMMB (μg mL^−1^)				
	0	50	100	150	200
Viable cells	89.96 ± 0.44%	83.72 ± 0.57%	81.68 ± 0.67%	71.59 ± 0.57%	11.43 ± 0.30%
Early apoptosis	1.24 ± 0.10%	2.07 ± 0.16%	2.02 ± 0.87%	2.22 ± 0.19%	1.32 ± 0.12%
Late apoptosis	8.15 ± 0.37%	13.56 ± 0.56%	15.30 ± 0.67%	23.52 ± 0.72%	81.02 ± 0.44%
Necrosis	0.66 ± 0.07%	0.66 ± 0.08%	1.00 ± 0.05%	2.66 ± 0.12%	6.24 ± 0.29%

The gated cell population represents the number of apoptotic A549 cells cultured in the medium containing DMMB after 2 days. Each value is expressed as the mean ± SD (*n* = 5).

## Abbreviations

KLEE: *C. kanehirai* leaf ethanol extract; KLWE: *C. kanehirai* leaf water extract; KFW: *C. kanehirai* floral water; PDA: potato dextran agar; JTA: Job’s tears agar; MEAC-KLEE: methanol extracts from *A. cinnamomea* cultured on KLEE; DPPH: 2,2-diphenyl-1-picrylhydrazyl; EC50: effective concentration; DMMB: 2,4-dimethoxy-6-methylbenzene-1,3-diol; HPLC: high-performance liquid chromatography; NMR: nuclear magnetic resonance; GAE: gallic acid equivalents; ME: methanol extract.
